# Two cases of focal status epilepticus in pregnancy

**DOI:** 10.1016/j.ebr.2021.100483

**Published:** 2021-09-15

**Authors:** Andrew Christiana, Micaela Della Torre, Anna Serafini

**Affiliations:** University of Illinois Hospital & Health Sciences System, 1740 w Taylor St, Chicago, IL 60612, USA

**Keywords:** Pregnancy, Focal status epilepticus, Fetal heart tracing, Epilepsy, Continuous EEG

## Abstract

The management of women with epilepsy (WWE) presents many challenges for physicians. The primary goal during pregnancy is to achieve the best possible control of seizures with the least adverse effects associated with exposure to antiseizure medications (ASMs). Even though the guidelines for managing pregnant WWE are expanding, no definitive guidelines exist for the treatment of status epilepticus (SE). Additionally, much of our data comes from the effect of generalized tonic clonic seizures on the fetus. There is very little data on the effect of focal seizures and even less on focal SE. Here we present two cases of pregnant WWE who presented in focal SE, who underwent simultaneous video-EEG monitoring and fetal heart tracing (FHT). During each focal seizure the FHT demonstrated a normal baseline heart rate with moderate variability. In the second case due to continuous seizures more aggressive treatment had to be started. This led to maternal relative autonomic instability as well as absent variability on FHT. These findings raise many questions on the management of focal SE during pregnancy including if the effect of treatment is worse than the seizures, and how do we balance our goals for both mother and fetus?

## Highlights

• Two pregnant women with a history of epilepsy were found in focal status epilepticus.
• VEEG and fetal heart tracing were examined simultaneously with the patient’s seizures.
• The patient in Case 1 was treated more conservatively than Case 2 despite similar findings on FHT.
• Case 1 eventually stopped seizing while Case 2 developed complications related to ASM treatment.
• How can we incorporate FHT during the management of pregnant women with seizures?

## 1 Introduction

Women with Epilepsy (WWE) are known to pose numerous challenges during pregnancy to both mother and fetus. The primary goal in the treatment of epilepsy during pregnancy is to achieve the best possible control of seizures with the least adverse effects associated with exposure to antiseizure medications (ASMs). ASMs are known to increase the risk of congenital malformations and affect the offspring’s cognitive outcome [1]. Seizure freedom during pregnancy is crucial as seizure deterioration during pregnancy is a serious complication that may affect maternal and fetal health. The majority of case reports deal with generalized tonic clonic (GTC) seizures as opposed to focal seizures. Direct complications of GTC seizures to the mother alone are understood and include sudden unexplained death from epilepsy [2] (SUDEP), brain injury due to neuronal death, and a significant decrease in quality of life. Additionally, maternal GTC seizures can cause direct injury or death to the fetus as a result of trauma [4]. There is little data available on the direct effects of focal seizures, and even less data on the effect of focal status epilepticus (SE). Here we report two cases of pregnant WWE who were found to be in focal SE and underwent simultaneous cardiotocography and continuous video EEG monitoring.

### 2.1 Case 1

29-year-old right-handed female G1P0 with past medical history of right temporal lobe epilepsy since age nine, who presented at 25 weeks and 5 days gestation, as a transfer from an outside hospital (OSH) for recurrent focal retained awareness seizures. Seizures were described as left arm clonic jerking and left eye deviation with preserved consciousness. Prior to the hospital admission, she underwent a mid-second trimester sonogram, which showed fetal agenesis of the cerebellar vermis with an enlarged cisterna magna, consistent with a Dandy-Walker variant. Her home medications prior to hospital admission were prenatal vitamins, folic acid 4 mg daily, levetiracetam 2000 mg twice daily, carbamazepine 400 mg twice daily, and phenobarbital 30 mg in the a.m. and 60 mg in the p.m. However, upon admission, patient reported taking half of the phenobarbital dose than she was prescribed. In the obstetrical emergency room she was immediately monitored to identify the presence of fetal heart tones (FHT) which was reassuring. Her vitals and labatory studies were unremarkable and no evidence of infection or pre-eclampsia was evident. Her ASM levels showed a normal carbamazepine level (4.3mcg/mL) but low levetiracetam (<10 mcg/mL) and phenobarbital (10.1 mcg/mL) serum conventration, suggesting the etiology of her seizures was secondary to non-compliance.

The patient underwent video-EEG monitoring which showed electrographic seizures arising from the right centroparietal area with spread to the right posterior temporal area every five minutes, consistent with focal non-convulsive status epilepticus (NCSE). Some of those seizures had a clinical correlate characterized by flexion and rhythmic jerking of the left upper limb with loss of awareness lasting 10–20 seconds. She was loaded with 1300 mg fosphenytoin overnight which decreased her seizure frequency to once an hour for only a few hours.

The next morning the patient was transferred to the Neurological Intensive Care Unit where she was administered phenobarbital 60 mg and fosphenytoin 100 mg three times daily while continuing the same doses of levetiracetam and carbamazepine. Over the next two days, her seizure frequency gradually decreased and became mostly subclinical until she was seizure free on day four. She was then transferred back to the antepartum service where she underwent an MRI brain without contrast which was unremarkable for new pathology. On hospital day five she was stable for discharge.

FHT and video-EEG were monitored simultaneously. FHT was reported daily as appropriate for gestational age with moderate variability, no accelerations, or decelerations. Two-hour neonatal stress tests were continued three times daily by obstetrics with no changes noted during her seizures (both clinical and subclinical), and ASM IV loading. Her cervix was checked daily and reported to be closed, thick, and acontractile. Her vitals remained stable with no episodes of desaturation or mean arterial pressure  < 65 mmHg. She was discharged home and later, at 39 weeks and 2 days, gave birth to a viable baby with APGAR scores of nine and nine.

**Fig. 1:**
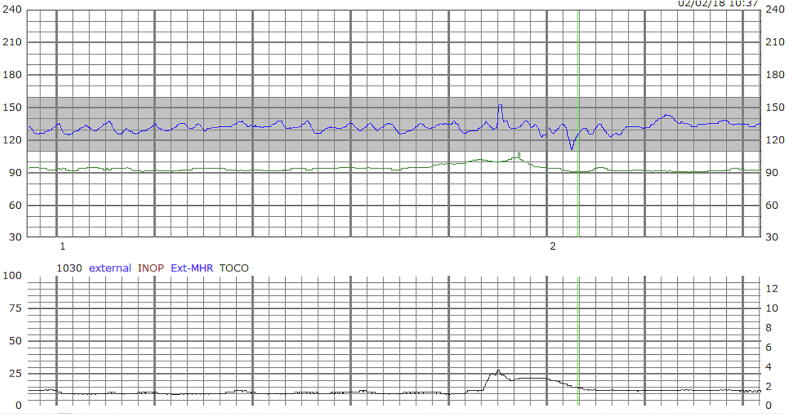

Fig. 1 shows the external cardiotocography of the patient in Case 1 during one of her seizures. The graph at the top demonstrates a baseline heart beat of 130 with moderate variability [(beats per minute (BPM) over time)]. The bottom graph demonstrates tocography with 1 contraction in a period of 7 minutes (mmHg over time).

**Fig. 2:**
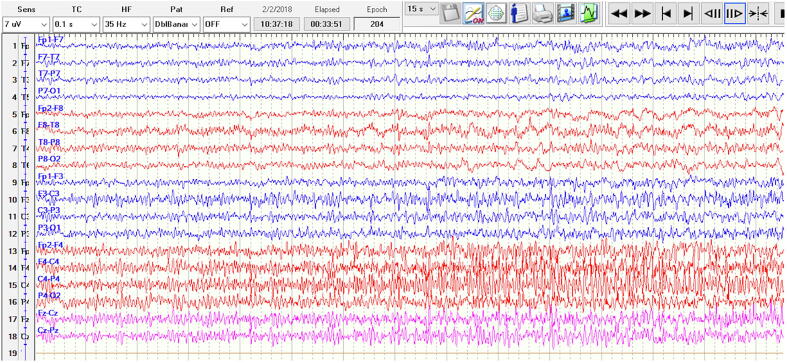

Fig. 2 shows the EEG during the FHT in Fig. 1. Using a anterior-posterior bipolar (“double banana”) montage, there is a buildup of fast activity in C4-P4 that evolves in frequency and spreads to F4. There is a further spread of rhythmic delta activity to T8-P8 toward the second half of the image.

### 2.2 Case 2

A 26-year-old female G3P1011 with a history of developmental delay, prior imaging demonstrating colpocephaly with ectopic gray matter in the bilateral occipital horns, and a history of focal epilepsy since age 11, was admitted at 27 weeks of gestational age in focal SE. Seizures were characterized by right head and eye deviation leading to right arm jerking with minimal responsiveness. Her home medications included phenytoin, phenobarbital, and levetiracetam. However, her phenytoin level was undetectable and after discussion with the patient it appeared she was only taking her levetiracetam. Labs and vitals were unremarkable for infection or pre-eclampsia. Phenytoin was loaded and restarted and levetiracetam was continued. Video-EEG was consistent with focal SE with seizures arising from left posterior quadrant. Most of her seizures had a clinical correlate as described above, with few subclinical episodes observed.

She was transferred to the Neuroscience Intensive Care Unit with a seizure frequency of once every three to eight minutes despite increasing levetiracetam to 2 grams BID, adding clonazepam 1 mg TID, and magnesium sulfate. With continued seizures, it was decided on day three at 11 pm to load with phenobarbital 600 mg. Unfortunately, no changes in seizure frequency were observed. In the morning of day four she was intubated and placed on propofol titrated up to 212 mg/hour resulting in a moderate reduction of seizures.

Initially the FHT was reassuring with a baseline 140 s, moderate variability, occasional accelerations, and no decelerations during all her seizures until the phenobarbital load and subsequent intubation. At this point she is documented to be relatively hypotensive with a decrease in heart rate from 90 s to 60 s and no hypoxemia. This persisted and mildly worsened with the initiation of propofol. Unfortunately, the exact temporal relationship was unclear. However, after the propofol was started FHT demonstrated absent/minimal variability with fetal bradycardia. Given she was still having frequent seizures and now progressing toward super-refractory SE, a multidisciplinary meeting was conducted between Maternal Fetal Medicine, Neonatology, Neurology, and Anesthesia where it was decided she would be taken for a cesarean section. The baby was subsequently delivered at 28 weeks and 0 days with Apgar scores of one at one minute, three at five minutes and four at ten minutes. The baby’s birth weight 1070 grams with no obvious deformities.

After the cesarean section she was transferred back to the Neuroscience Intensive Care Unit where propofol was discontinued and midazolam was added to phenytoin, levetiracetam, and phenobarbital. Video-EEG now demonstrated burst suppression pattern for two hours and then diffuse delta slowing without underlying epileptic activity. Over the next two days midazolam was weaned off and she was extubated. Over the four days post-operatively her EEG gradually improved to diffuse theta slowing with the development of sleep architecture. Her clinical exam improved and on the day of discharge as she was alert and oriented without focal deficits.

## 3 Discussion

The primary goal in the treatment of epilepsy during pregnancy is to achieve the best possible seizure control with the least adverse effects associated with exposure to ASMs. Various numbers have been reported but it is suggested that only between 48 to 60% of WWE are seizure-free in pregnancy [3]. The variation is likely due to numerous factors including metabolic changes, ASMs concentrations, ASMs compliance, seizure etiology, and the most reliable being pre-pregnancy seizure control [3]. Seizures are known to affect maternal and fetal health, however, the relative impact of different seizure types is difficult to determine. Most data are on maternal GTCs where it is commonly believed that they could lead to fetal hypoxia through decreased placental blood flow, post-ictal apnea, or academia [4]. Teramo et al reported two case reports of pregnant women with GTCs. One patient was cyanotic appearing briefly postictally while the FHT showed a 13-minute continuous bradycardia wave with decreased short-term variability [5]. Afterward there was a short phase of tachycardia with short-term and long-term variability. The second case noticed similar, yet shorter, findings [5].

Less data is available on the effect of focal seizures. At least one prior case report has demonstrated a fall in fetal heart rate after a brief event associated with transient maternal hypoxia [6]. Another report by Sahoo and Klein refers to a patient who experienced focal seizures secondary to cavernous hemangiomas while seven months pregnant [7]. During the seizure, the fetal heart rate fell from 160 to 70 bpm and returned to baseline two minutes after the seizure. Maternal autonomic instability was also seen with oxygen saturations down to 75% and a concomitant heart rate of 125 that improved after her seizure [7]. Aside from autonomic instability, the direct effect of focal seizures on placental blood flow, fetal acidemia, and other factors remains unknown. Part of the challenge is that a “focal” seizure encompasses a wide variety of phenotypical presentations.

Even less data is available for fetal changes during SE. In pregnancy, SE is rare and may occur during gestation, labor, or puerperium [8]. Here we reported two cases of focal SE during which we had the advantage of correlating video-EEG data simultaneously with FHT. For both women the cause of SE was medication non-compliance with poor pre-pregnancy seizure control. Case 1 EEG showed frequent clinical and subclinical seizures originating from the right centroparietal region. Neonatal stress tests were conducted three times a day, two hours each time, over 3 days of hospital admission. Consistently, the FHT showed a normal baseline with moderate variability consistent with early gestational age. The seizures on EEG were correlated to the FHT and no changes were observed during both clinical and electrographic seizures. Case 2 EEG demonstrated seizures arising from the left posterior temporal region and subsequently resulted in super refractory status-epilepticus. Throughout three days of monitoring her FHT demonstrated 120–140 s bpm with accelerations and without decelerations. It wasn’t until being loaded with phenobarbital and subsequently intubated on propofol that the FHT started to demonstrate absent/minimal variability with a baseline heart rate 120 bpm. Since FHT were consistently reassuring until this moment, the etiology is likely the ASMs and not the seizures directly.

Highlighting the difference in management between Case 1, minimal ASM escalation, versus Case 2, significant ASM escalation, poses important questions the treating neurologist should consider. How can we estimate the potential risk of the seizure on both the mother and the fetus? And importantly, does our priority lie in controlling the maternal seizure or ensuring the health of the fetus when we find these goals are in contradiction? In our second case, per FHT, the focal seizures did not seem to pose immediate danger to the fetus. Changes were seen only once sedation was started. Though definitive signs of fetal distress such as decelerations were not present, the FHT was trending toward a concerning pattern. Whether the cause was relative maternal autonomic changes or the direct effect of propofol crossing the placenta [9] remains uncertain. Situations such as these pose an ethical dilemma where more information provided by our various monitoring techniques could help us answer. More importantly, we could guide a more informed decision during discussions with our colleagues, patient, and family. In the meantime, we continue to work in a grey area by balancing the effect of medications and emergent delivery against the direct effect of continuous focal seizures on both the mother and fetus.

## 4 Conclusion

In conclusion, more data is needed to better understand the optimal management of pregnant patients in focal status epilepticus. The management remains complicated, but a larger trial that includes FHT may provide information and potentially affect management guidelines.

## Ethical statement

In this manuscript both patients consented to treatment. Both patients’ confidentiality has been maintained and no patient identifiers have been reported or documented. All authors on this paper have made a significant contribution to this manuscript. There were no competing interests involved.

## Declaration of Competing Interest

The authors declare that they have no known competing financial interests or personal relationships that could have appeared to influence the work reported in this paper.
